# Neurodevelopmental effects of anesthesia and environmental factors

**DOI:** 10.18632/oncotarget.14694

**Published:** 2017-01-17

**Authors:** Anatoly E. Martynyuk, Jian-Jun Yang, Jia-Qiang Zhang

**Affiliations:** Department of Anesthesiology and the McKnight Brain Institute, University of Florida College of Medicine, Gainesville, FL, USA

**Keywords:** brain, development, general anesthetic, GABA, stress, epigenetic, Neuroscience

The question of whether early life exposure to general anesthesia induces neurodevelopmental abnormalities in humans, similar to what has been demonstrated in animals from rodents to rhesus monkeys, is complex and of significant clinical concern, given that more than 1 in 4 children undergo procedures requiring anesthesia in their first year of life [1]. Important roadblocks to understanding the developmental outcomes of human exposure to general anesthetics include poor understanding, even in rodents, of the full spectrum of developmental abnormalities, their underlying mechanisms, and their modulation by subsequent life experiences.

Rodents become largely resistant to the side effects of general anesthetics by the end of the second postnatal week. The existence of a vulnerable period suggests that prime targets for modulation by general anesthetics are mechanisms unique to this period of life in rodents. One of the unique features of the rodent brain during the first 2 postnatal weeks is relatively high and low expressions of the neuronal Na^+^-K^+^-2Cl^-^ Cl^-^ importer and the K^+^-2Cl^-^ Cl^-^ exporter, respectively. As a result, the transmembrane gradients for Cl^-^ support outward depolarizing Cl^-^currents through gamma-aminobutyric acid type A receptor (GABA_A_R) channels. This GABA_A_R-mediated depolarization is sufficient to activate calcium influxes that play a crucial role in regulation of many developmental processes, including neurogenesis and synaptogenesis. Abnormal GABA_A_R-mediated depolarization, however, has been linked to a number of developmental disorders, including autism spectrum disorders and schizophrenia. Most general anesthetics enhance GABA_A_R-mediated currents. Interestingly, the ontogenetic transition in GABA_A_R-mediated signaling from stimulatory to inhibitory coincides with the age period when rodents become resistant to developmental effects of GABAergic anesthetics.

During the first 2 postnatal weeks, rodents undergo a stress hyporesponsive period (SHRP) during which the limbic-hypothalamic-pituitary-adrenal (LHPA) axis — the main stress response system — is hyporesponsive to stressful stimuli. The SHRP is believed to be an important adaptive feature to protect the developing brain from deleterious effects of high levels of stress-related hormones, such as corticosterone. Stressful stimuli that are sufficient to overcome the SHRP, such as prolonged and repeated maternal separations frequently used for this purpose in laboratory settings, lead to neurobehavioral abnormalities and exacerbated endocrine responses to stress in adulthood. The LHPA axis and GABA_A_R activities are closely linked. For example, neuroactive steroids, released by the adrenal gland in response to stress-induced stimulation of the LHPA axis, down-regulate LHPA axis activity by enhancing GABA_A_R-mediated inhibition. It is plausible that in the neonatal brain, neuroactive steroids or GABAergic anesthetics, by enhancing GABA_A_R-mediated depolarization, exacerbate or induce stress-like responses.

In support of such possibility, we found that sevoflurane and propofol, two general anesthetics that share their abilities to enhance GABA_A_R activity, could “overcome” the SHRP in neonatal rats. Thus, single exposure of neonatal rats to sevoflurane or propofol caused multifold increases in systemic corticosterone levels and electroencephalography-detectable seizures at the time of anesthesia [2–8]. In adulthood, the exposed rats exhibited altered hippocampal synaptic activity, impaired sensorimotor gating function, deficient memory during the Morris water maze and fear conditioning tests, anxiety-like behavior, heightened levels of corticosterone at rest, and exacerbated corticosterone responses to physical restraint. Many of these effects were diminished by the Na^+^-K^+^-2Cl^-^inhibitor bumetanide, suggesting that anesthetic-enhanced GABA_A_R-mediated depolarization may be one of initial steps in mechanisms that program anesthetic-induced developmental abnormalities.

The exacerbated corticosterone responses to acute stress months after exposure to sevoflurane or propofol suggest that stressors in post-anesthesia life can further potentiate developmental abnormalities initially programmed by GABAergic anesthetics. This possibility is supported by our findings that rats neonatally exposed to sevoflurane and then housed from the time of weaning in isolation in enrichment-deprived environments had behavioral abnormalities as well as reduced levels of brain-derived neurotrophic factor, synaptic protein markers, and survival of new granule cells in the hippocampus, while those exposed to sevoflurane and housed in groups in enriched environments were not different from not-exposed counterparts [7]. These findings further support the possibility that GABAergic anesthetics administered during early period of life can be considered an environmental factor that predisposes the subject to stress vulnerability later on. Identification of such factors and the mechanisms underlying their effects is of major clinical and basic neuroscience concern, as dysregulated stress response systems have been linked to the pathophysiology of a number of neuropsychiatric disorders.

The epigenetic mechanisms, such as DNA methylation and histone acetylation, are thought to be involved in mediation of environmental factor-induced shaping of the developing brain. We have found that, when administered to rats neonatally exposed to sevoflurane, the histone deacetylase inhibitor sodium butyrate normalized hippocampal density of dendritic spines, levels of brain-derived neurotrophic factor, phosphoCREB, synaptic protein markers, acetylated histones H3 and H4, and histone deacetylases 3 and 8, as well as rats’ behavior, suggesting a potential role of epigenetic mechanisms as one of the mediators in the developmental effects of general anesthetics [8].

Environmental enrichment that may prevent anesthetic-induced developmental abnormalities in otherwise healthy rodents is not an issue for the majority of human patients because this is typical for most children. If similar mechanisms are operative in humans, then development in healthy human patients who experience normal stress levels may be minimally if at all affected after exposure to general anesthesia. However, most children who require general anesthesia during the early postnatal period inevitably experience a variety of stressors during post-anesthesia life (e.g., diseases, pain, hunger, psychological stress). Such patients may be at risk of developing early life anesthetic-programmed neuroendocrine and neurocognitive abnormalities.
